# Psychiatric referrals in general practice

**DOI:** 10.1192/j.eurpsy.2021.1079

**Published:** 2021-08-13

**Authors:** M. Turki, T. Babbah, R. Ouali, S. Ellouze, W. Abid, R. Charfi, N. Halouani, J. Alouou

**Affiliations:** Psychiatry “b” Department, Hedi Chaker University hospital, sfax, Tunisia

**Keywords:** general practice, psychiatrists, referrals, Mental disorders

## Abstract

**Introduction:**

Over half of patients with mental disorders are seen by primary care physicians. However, as for patients with somatic problems, referral to psychiatrists seems to be sometimes necessary.

**Objectives:**

The present study aimed to identify reasons and difficulties perceived by general practitioners (GP) in mental health referrals.

**Methods:**

A cross-sectional web-based survey was conducted between August 22 and September 23, 2020, so that 47 responses of GP were included.

**Results:**

The mean age of respondents was 37.3 years. Their seniority as doctors was 8 years on average. Among them, only 17% attended a post-university psychiatric training. The participants reported that they refer on average 32.5% of patients with mental disorders to psychiatrist: 85.1% to psychiatric hospital, 40.4% to liberal psychiatrists and 21.3% to clinical psychologists. Regarding the reasons for referral to mental healthcare structures, 70.2% of doctors justified their doing so by their insufficient training in mental healthcare; 66% by a need for hospitalization, 57.4% by the presence of delusions, while in 27.7 % of cases, the transfer was carried out at the request of the patient or his family. The difficulties mentioned by GP were patient refusal to consult a psychiatrist (70.2%) and difficulties related to the management delay (44.7%).

**Conclusions:**

Patient and health system factors, as well as physicians experience seem to have important influences on mental health referral. Open communication and ease of consultation with psychiatrists can make the care of patients with mental health problems even more rewarding to the primary care physician.

